# Characterization of Factors Predicting a Favorable Opinion of Research Applications Submitted for an Ethical Review Process

**DOI:** 10.3389/fmed.2022.878786

**Published:** 2022-06-16

**Authors:** Eduardo Mirpuri, Lara García-Álvarez, María Teresa Acín-Gericó, Blanca Bartolomé, Roberto C. Delgado Bolton, Montserrat San-Martín, Luis Vivanco

**Affiliations:** ^1^Research Ethics Committee of La Rioja (CEImLAR), Rioja Health Foundation, Logroño, Spain; ^2^Unit for Clinical Research Support, Center for Biomedical Research of La Rioja (CIBIR), Logroño, Spain; ^3^Subdirectorate of Pharmacy and Provisions, Navarre Health Service, Pamplona, Spain; ^4^Platform of Bioethics and Medical Education, Center for Biomedical Research of La Rioja (CIBIR), Logroño, Spain; ^5^National Centre of Documentation on Bioethics, Rioja Health Foundation, Logroño, Spain; ^6^Department of Diagnostic Imaging (Radiology) and Nuclear Medicine, University Hospital San Pedro, Logroño, Spain; ^7^Department of Statistics and Operational Research, University of Granada, Melilla, Spain; ^8^Scientific Computing & Technological Innovation Center (SCoTIC), University of La Rioja, Logroño, Spain

**Keywords:** Research Ethics Committee (REC), research applications, predictors, informed consent, leadership and mentoring, research methodology and ethics, COVID-19, Spain

## Abstract

**Introduction:**

In Spain, biomedical research applications must receive a positive ethical opinion from Research Ethics Committees (RECs) before being executed. There is limited information on how to optimize the ethical review process to reduce delays. This study was performed to characterize variables predicting favorable opinions at the first ethical review performed by a REC.

**Material and Methods:**

The study assessed all research applications revised by a REC in 2019–2020. Data was extracted from REC's database of La Rioja, Spain. Variables collected covered three areas: (i) principal investigator's profile; (ii) study design; and (iii) ethical review process. A model based on multiple logistic regression analysis was created to identify variables explaining favorable opinions in first rounds of ethical review processes.

**Results:**

The sample included 125 applications (41 submitted in 2019, and 84 in 2020). At the first review, nine (7%) applications were rejected, 56 (45%) were approved, and the remaining 60 (48%) required at least two reviews prior to approval. When comparing both years, a 2-fold increase in the number of applications submitted, and a difference in the ratio of applications with a favorable vs. non-favorable opinion were observed. Furthermore, a model predicted 71% of probability of obtaining a favorable opinion in the first ethical review. Three variables appeared as being explanatory: if the principal investigator is either the group leader or the department's head (OR = 17.39; *p* < 0.001), and if the informed consent (OR = 11.79; *p* = 0.01), and methods and procedures (OR = 34.15; *p* < 0.001) are well done.

**Conclusions:**

These findings confirm an increase in the number of submissions and a difference in the ratio of applications approved by year. Findings observed also confirm deficiencies in “informed consent” and in “methods and procedures” are the two main causes of delay for favorable ethical opinions. Additionally, findings highlight the need that group leaders and heads of departments should be more involved in guiding and supervising their research teams, especially when research applications are led by less experienced researchers. Based on these findings, it is suggested that an adequate mentoring and targeted training in research could derive in more robust research applications and in smoother ethical review processes.

## Introduction

Patients play an essential role in biomedical research, either as study subjects or as source of clinical data. Hence, it is expected that all professionals actively involved in biomedical research – in addition to the necessary technical, clinical and scientific knowledge – should have an individual commitment with professionalism in order to work in consonance with the ethical and legal frames that accompany their research activities (1). This professionalism is synonym of a “job well done” placing a high value of doing a good job, as well as respecting the autonomy of the patients and acting with integrity.

### Research Ethics Committees as Guarantors of Professionalism in Biomedical Research

The main responsibility of a Research Ethics Committee (REC) is to guarantee that a biomedical research application meets the standards of scientific, ethical and legal rigor prior to its experimental execution (2). This obligation, in Spain, has been regulated within the framework of the Organic Law (*Ley Orgánica*, LO) LO 3/2018 (3), both on the protection of personal data; the LO 41/2002, on patient autonomy and obligations regarding information and clinical documentation (4); the SCO/362/2008, on good clinical practice (5); and the Royal Decree (*Real Decreto*, RD) RD 1090/2015, on clinical trials with drugs (6). Therefore, shortcomings in any aspect related to methods, procedures, ethics or legal frame could lead to a reevaluation, or even rejection by the REC responsible to review a given research application. Nevertheless, reaching this goal implies that the REC must be composed by a robust structure capable to address issues coming from different disciplines (7).

Despite the existence of different national normatives, there are certain coincidences in common aspects of the RECs' activity that could be improved. For example, McNeill (8), Beshir (9), and Wagner and colleagues (10) agree that the REC's administrative work involves a slow bureaucratic process, which has been sometimes criticized as costly or at least making the review process laborious. Others have criticized that inflexible requirements for adherence to narrow literal interpretations of certain normative can lead to a system that is more concerned with “legalism” than the protection of human research participants (2, 11). Furthermore, some authors (12, 13) have highlighted the inconsistencies across different committees, even though they were following the same national normative. In this regard, Edwards and colleagues (14) argue that those inconsistencies are negative only when they derive from a lack of expertise in identifying ethical issues in the research applications that are revised. In addition, Beshir (9) remarks that it is crucial for a REC to ensure that researchers have sufficient research experience and qualifications or alternatively are collaborating with experienced colleagues in the field of their research. This is especially important in two circumstances: when research procedures imply risk for researchers, participants or the environment; and when sensitive aspects related to the privacy and patients' identity can be exposed. In both cases, it is a duty of RECs to minimize the risk of any harm. Finally, some authors have suggested to reassess the role of RECs in order to ensure their purpose is fulfilled to encourage the scientific development maintaining an acceptable ethical framework (7). However, in practice, reaching this goal does not look feasible due to the implicit nature of the ethics and the deliberative methodology followed by the RECs.

The situation above described became more stressful in the last 2 years due to the rapid expansion of the SARS-CoV-2 pandemic and the extremely high number of SARS-CoV-2 infections (COVID-19). The pandemic not only tested the capacity of the scientific community for finding therapeutic and preventive responses to contain and mitigate the disease, but also derived in a greater effort for RECs to guarantee that the increasing number of research applications in different areas were in accordance with scientific, ethical and legal standards. Aware of this situation, the World Commission on the Ethics of Scientific Knowledge and Technology (COMTEST), and the International Bioethics Committee (IBC) signed a joint Ethical Declaration entitled “Ethical considerations from a global perspective”, 2 months after the pandemics was declared (15). The aforementioned document has been received from the research ethics committees as an important exhortation to establish actions in order to facilitate a rapid scientific and technological development, but without neglecting the ethical standards that should always frame any research activity involving human subjects (16). In consonance with this, the Bioethics Committee of Spain (*Comité de Bioética de España*, CBE) prepared a Report with ethical and legal guidelines that researchers working with health data and biological samples should fulfill during the pandemic (17). In addition, the Spanish Agency of Medicines and Medical Devices (*Agencia Española de Medicamentos y Productos Sanitarios*, AEMPS) elaborated a special core of guidelines for research activities involving either patients infected with COVID-19 or clinical data from these patients (18). As receptor of these guidelines, each REC was in charge of conciliate practical needs with ethical principles in an extraordinary socio-sanitary context with new research projects increasing rapidly in time.

### Study Purpose

Evidence of the effect that the pandemic situation has had and in still having on the RECs is scarce. In Spain, there is only one study performed by the REC of the Autonomous Community of Galicia (19). Bugarin-Gonzalez and colleagues, authors of the aforementioned study, reported that the vast majority of research applications submitted to their REC met the ethical requirements necessary for their approval. However, more than a third of them included deficiencies either in methodological aspects or in the informed consent. According to them, these deficiencies could be associated with a lack of knowledge in the normative, training gaps related to management in biomedical research, and a poor communication and interdisciplinary collaboration in the research teams, especially with more experienced professionals. These deficiencies derived not only in delays in the start of research activities, but also implied a work overload in the REC that was forced to dedicate more than one round of revisions for the same application prior to giving a final approval.

Based on the situation described above, this study was designed with the purpose of confirming the following hypothesis: Research group leaders and department heads are usually the most experienced researchers in their groups. In consequence, they play an important role in the success of research applications performed by their teams as first guarantors of a “job well done.” This involvement can be reflected in a favorable opinion of a REC, once those applications are submitted for a first ethical review. With this purpose, four research objectives were pursued:

To collect information of the research applications submitted for an ethical review process based on three areas: profile of the researcher who led the study (principal investigator, PI), aspects related to the study design, and aspects related to the ethical review process.

To identify the ratio of research applications that obtained a favorable opinion in the first ethical review and to confirm whether this ratio was different in the 1st year of pandemic in comparison with the year before the initiation of the pandemic.

To analyse which of the aforementioned variables showed statistical association with a favorable opinion in the first ethical review.

To characterize which of the variables with statistical association appear as predictors of a favorable opinion in the first ethical review.

## Materials and Methods

### Study Sample

This study covered the 125 research applications submitted to the Research Ethics Committee of La Rioja (*Comité Ético de Investigación con medicamentos de La Rioja*, CEImLAR), between January 1st, 2019 and December 31st, 2020. Only applications referred to biomedical research projects were included. Other types of projects, such as clinical trials with previous ethical favorable opinion from another REC (REC of Reference), and informative post-authorization studies, were not included in this study. According to the Royal Decree 1090/2015, which regulates clinical trials with medicines, the Ethics Committees of Drug Research and the Spanish Registry of Clinical Studies, clinical trials following a multicentre study design only require the ethical evaluation of one REC, which will be the REC of Reference.

The CEImLAR was initially established as CEICLAR (*Comité Ético de Investigación de La Rioja*) in 1995 by Order 10/1995, March 2nd of the Autonomous Community of La Rioja. This Order was updated in 2005 (Order 71/2005, December 2nd). In 2018, the CEICLAR received a certification from the Regional Ministry of Health of La Rioja as a Research Ethics Committee with drugs (CEImLAR). Following the current Spanish normative, the main objective of the CEImLAR is to guarantee the protection of human rights, safety, and well-being of participants and the society as a whole in the framework of activities related to the clinical research, health and scientific advances in La Rioja. Thirteen members, including a permanent secretariat with voice but not vote, currently comprise the CEImLAR. Nine of these members are healthcare professionals (including specialists on clinical and primary care pharmacology, pharmacy, medicine, and nursing), two members are professionals from other disciplines different from medicine (including a lawyer with specialization in data protection, and an economist), a member from a patients' association, and a member with specialization in bioethics. In addition, different regional public health organizations must be represented in the committee's structure. Similar as others RECs, the current activity of the CEImLAR is focused in the evaluation of the methodological, ethical and legal issues of any biomedical research intended to be performed in La Rioja, according to the evaluation criteria stablished by the national normative.

### Main Measures

Variables collected were distributed in three groups. Variables composing the first group included information related to the characteristics of the principal investigator (PI), defined as the researcher who led the study and submitted the application. These variables were: identity and academic background of the PI, if the PI was alone or had a research team, if the PI was a professional-in-training (in those cases where the application was part of a post-graduate training program and the PI had a mentor or tutor), and position of the PI in his/her research group, department or unit. The second group of variables referred to different aspects related to the study design. These variables included: the type of study (observational or interventional), the research design (retrospective or prospective), the methodology applied (quantitative, qualitative, or both), participants recruited (minors, adults, or both), and usage of an informed consent form. Finally, aspects related to the ethical review process were collected in a third group of variables. These variables were: dates of first and final ethical revision, time-span (days) between the first and the final opinion of the REC, first and final opinion, number of clarifications required, and type of deficiencies reported in the first ethical review process. Since such deficiencies were not classified in the reports performed by the REC, six categories were created: “informed consent,” “objectives and/or hypotheses,” “methods and/or procedures,” “legal aspects,” “conflict of interests,” and “economic aspects.” Examples of deficiencies reported in the “informed consent” category were applications with informed consent forms in poor writing, with confusing information, or with missing sensitive information. Category “objectives and/or hypotheses” referred to following type of deficiencies: applications with poor writing or confusing research objectives or hypotheses, or applications where those aspects were not connected with the procedures or methodological aspects previously described. Poor writing procedures or procedures missing sensitive aspects related to the process of participants' recruitment, data manipulation, or techniques planned for application in the research protocol, were examples of deficiencies reported in “methods and/or procedures” category. Examples of deficiencies included into the “legal aspects” category were applications not using updated normative or that were not in accordance with the current normative. Applications with financial or other personal considerations that the REC considered could compromise (or had the appearance of compromising) the research purpose and were not reported by the principal investigator, were included in the “conflict of interest” category. Finally, applications requiring clarifications in relation to the sources of financial support, or applications inquired to bring information related to the budget or financial source, were included in the “economic aspects” category.

### Procedures and Ethical Approval

Data were extracted from the main database of the CEImLAR. This preliminary search covered all research applications presented in 2019 and 2020 based on the inclusion/exclusion criteria and in the pre-defined categories mentioned above. Three researchers, who are members of the CEImLAR (EMM, LGA, and MTAG), extracted the information and created a preliminary dataset. The identity of the PIs was collected in order to search for two indicators of scientific productivity (articles published and h-index) from SCOPUS. Identities of the PIs were extracted in a second dataset after the preliminary dataset was pseudonymized with alphanumeric codes. Another researcher (BBC), who was not a member of the CEImLAR, completed a second dataset with the information collected from SCOPUS. Finally, both datasets were merged. This procedure was performed with the purpose of keeping anonymous the identity of the PIs. The Research Ethics Committee of the Foral Community of Navarra, an independent Research Ethics Committee, approved the aforementioned procedure prior to be executed (Ref. PI 2021/57).

### Statistical Analysis

The opinion submitted by the REC after a first ethical review was used as dependent variable. This variable was categorical and included four possible answers: “application rejected,” “application requiring clarifications,” “application approved with minor clarifications,” and “application approved.” For analysis purposes, this variable was recoded into a new binary one with two possible outcomes: zero (“failure”), when the application was rejected or required clarifications after the first ethical review was finished; and one (“successful”), when the application was approved with or without minor clarifications. All the other elements collected were treated as independent variables.

Chi-square test for nominal independent variables and the Mann–Whitney *U* test for quantitative independent variables were applied in bivariate analyses. Then, in a binary logistic regression model, the magnitude of association of the independent variables that showed a significant relationship in the previous bivariate analysis was determined. In order to measure the power of explanation of the logistic regression model obtained, the Nagelkerke's R squared was calculated. Finally, the weight of association between the dependent variable and its predictors was calculated by the measurement of the Odds ratio.

All analyses were done in the R language and programming environment for statistical and graphical analysis, version 3.6.2 for Windows and with the help of the statistical analysis packages *fmsb* (20), *nortest* (21), *rstatix* (22), and *OddsPlotty* (23).

## Results

The first objective was to collect information related to the PI, the research application, and the ethical review process. Analysis of SCOPUS database showed a range of publications between zero to 316 (M = 44; Mdn = 5; *SD* = 87.79). In the entire sample, 33 PIs did not have any article published in a peer-review journal, while the other 92 researchers had at least one article published. In relation to h-index, analysis showed a range of scores between zero to 40 (M = 9; Mdn = 1; *SD*=13.63). In the entire sample, 52 PIs had an h-index equal to zero, while the other 72 had h-indexes equal or higher than one. Also, analysis of the CEImLAR records showed that the time-span of the entire ethical review processes (from the 1st submission until the final opinion) ranged from zero to 550 days (M = 51; Mdn = 5; *SD* = 118.08). Distribution of the other characteristics related to the PIs, the research applications, and the ethical review process are summarized in the Table 1.

**Table 1:** Sample characteristics of categorical variables (*n* = 125).

**Variables**	**No (%)**	**Yes (%)**
*Principal investigator*		
Was a professional-in-training (the application was part of his/her training)	105 (84%)	20 (16%)
With research as his/her principal working activity	107 (86%)	18 (14%)
Discipline: medicine	37 (30%)^a^	88 (70%)
Academic degree: Doctoral or PhD	77 (62%)^b^	48 (38%)
Gender: male	65 (52%)	60 (48%)
With previous experience submitting a research application	61 (50%)	61 (50%)
Was the group leader or chief of his/her department	90 (73%)	33 (27%)
Was working alone	97 (81%)^c^	23 (19%)
*The study*		
Study type: observational	23 (19%)^d^	100 (81%)
Design: prospective	47 (38%)^e^	76 (62%)
Methodology: quantitative	13 (11%)^f^	110 (89%)
Required the use of an informed consent form	34 (27%)	90 (73%)
Participants: only adults	15 (12%)^g^	109 (88%)^h^
*Deficiencies reported at the first ethical review process*		
Documentation incomplete	105 (84%)	20 (16%)
Informed consent form	104 (83%)	21 (17%)
Hypothesis and/or research objectives	113 (90%)	12 (10%)
Methods and/or procedures sections	62 (50%)	63 (50%)
Legal aspects	106 (85%)	19 (15%)
Funding and economic report	110 (88%)	15 (12%)
Conflict of interest	125 (100%)	0 (0%)

a *Nursing (18), biology (5), biochemistry (2), chemistry (6), biotechnology (4), and pharmacy (2)*.

b *Master (21), Medical specialty (33), and Bachelor (23)*.

c *Research teams ranged between two to 10 members (IP included)*.

d *Interventional studies using a technical dispositive (3), a substance (4), and studies without using neither a dispositive or a substance (16)*.

e *Retrospective studies*.

f *Studies based on a qualitative methodology*.

g *Studies with only minors (5), and with minors and adults (10) as participants*.

h *Studies with patients (80), with healthcare professionals (10), and general public (19) as participants*.

The second objective was to identify the ratio of research applications with a favorable opinion in the first ethical review; and whether this ratio changed in 2020 in comparison with 2019. From the 125 applications analyzed, nine (7%) were rejected at the first ethical review, 60 (48%) required clarifications, nine (7%) were approved with minor clarifications, and 47 (38%) obtained an approval. Based on these findings, 56 (45%) research applications with approval or approval with minor clarifications were recoded as “successful,” while the other 69 (55%) were recoded as “failure.” The analysis of the entire review process showed that seven applications initially rejected, and other 55 applications requiring clarifications obtained a final approval after further reviews. Only five applications requiring clarifications were abandoned by their PIs without answering the queries performed. A comparative analysis by year showed, in one hand, a two-fold increase in the total number of applications submitted from 2019 to 2020. While in the 1st year 41 applications were submitted, 84 were submitted in the 2nd year. On the other hand, statistical differences appear in the ratio of applications with a favorable opinion over those with non-favorable opinion by year (χ^2^ = 4.23; *p* = 0.04). While this ratio was 0.46 in 2019, it changed into 1.05 in 2020.

The third objective was to determine whether a favorable opinion and each variable studied were statistically associated. Chi-squared tests confirmed a significant relationship between a favorable opinion and each of the following variables: PI with research as his/her principal working activity, PI with a Doctoral degree, being a male, PI with previous experience submitting a research application, and PI who is the group leader or the chief of his/her department. In addition, each variable related to different type of deficiencies reported in the first ethical review showed a statistical relationship with the judgement of the REC in the first ethical review. A summary is presented in Table 2. This relationship was not confirmed in the following cases: PI is a professional-in-training (and the application is part of his/her training), PI is a physician, and PI is working alone. Moreover, none of the second group of variables referred to aspects related to the study design were associated with a favorable ethical opinion. In addition, Mann–Whitney *U* tests confirmed that research applications with a favorable opinion were submitted by PIs with a higher number of publications (*p* < 0.001), and a greater h-index (*p* < 0.001).

**Table 2:** Frequency table of the REC's opinion in the first ethical review by variables collected.

**Variables**	**FO**	**NFO**	**χ^2^**
*Principal investigator*		
With research as his/her principal professional activity	12	6	4.07^*^
No	44	63	
Academic degree: Doctoral or PhD	29	19	7.68^**^
No	27	50	
Gender: male	33	27	4.85^*^
Female	23	42	
With previous experience submitting a research application	36	25	10.77^**^
Without previous experience	18	43	
Was the group leader or head of his/her department	28	5	29.39^***^
No	27	63	
*Deficiencies reported at the first ethical review process*		
Documentation incomplete	0	20	19.32^***^
No	56	49	
Informed consent	2	19	12.70^***^
No	54	50	
Hypothesis and/or objectives	0	12	10.78^**^
No	56	57	
Methods and/or procedures	6	57	63.92^***^
No	50	12	
Legal aspects	1	18	14.16^***^
No	55	51	
Economic aspects	1	14	10.02^**^
No	55	55	

*FO, favorable opinion; NFO, non-favorable opinion; χ^2^, Chi-squared coefficient;*

* *p < 0.05;*

** *p < 0.01;*

*** *p < 0.001*.

Finally, the fourth objective was to characterize which of the variables identified in the previous objective played a role as predictors of a favorable opinion. An analysis based on logistic multiple regression allowed to create a model predicting 71% of probability of obtaining a favorable opinion in the first ethical review (Nagelkerke *R*^2^ = 0.71). Three variables appeared as being explanatory in this model: if “methods and/or procedures” were complete (*p* < 0.001); if “the PI was the group leader or the head of his/her department” (*p* < 0.001), and if “informed consent” was well done (*p* = 0.01). A complete summary of these findings is reported in Table 3. From the analysis of the reports emerged the following common deficiencies in relation to “methods and/or procedures”: methods or procedures that were either poor writing or confusing, lack of information of participants' recruitment process, and ambiguous or missing information of data manipulation process. Regarding “informed consent,” three main types of deficiencies were observed: lack of informed consent form (due to researchers considered not necessary), contact information of the PI not included in the inform consent form, informed consents form with obsolete normative or normative not applicable to the study. In addition, a few informed consent forms either presented information overload, or were poor writing (text confusing, very technical or difficult to understand for the patient).

**Table 3:** Multiple logistic regression model.

	**β**	***SE***	**95% CI**	**OR**	***p***
Deficiencies in methods and/or procedures sections	+3.53	0.63	10.9–136	34.15	<0.001
The PI is the group leader or head of the department	+2.86	0.84	3.85–111	17.39	<0.001
Deficiencies in the informed consent	+2.47	1.00	2.06–121	11.79	0.01

*β, logistic regression coefficient; SE, standard error; CI, confidence interval; OR, Odds ratio; p, probability*.

*Nagelkerke R^2^ = 0.71*.

## Discussion

One of the aims of this study was to compare the number of applications submitted for an ethical review process in the 1st year of the pandemic and the ones submitted in the previous year. The findings observed confirm an increase in the number of submissions, and in the ratio of applications approved each year. Findings related to an increasing number of applications submitted are in accordance with the ones previously reported by the REC of Galicia (19). The difference reported in the ratio of approvals observed in this study can be explained by a certain grade of flexibility in the criteria followed by the REC in the ethical review process once the pandemic started. This flexibility is consequence of following the recommendations suggested by the AEMPS (18) under the extraordinary circumstances being suffered at the beginning of the pandemic.

Another aim was to identify which of the three group of variables collected (those related to the principal investigator's profile, aspects related to the study design, and aspects related to the ethical review process) were individually associated with a favorable opinion in the first ethical review. Findings observed in binary analyses confirmed a higher ratio of success when either the PI has research as his/her main professional activity, has previous experience preparing a research applications, is a male, or when there is a coincidence in which the PI is also the research group leader or the head of the department. Findings reported in this study indicate that neither having a doctoral or PhD degree (which should imply certain research experience), having a professional background different from medicine, nor preparing a research application in collaboration with other colleagues, were associated with a favorable opinion in the first ethical review. In addition, none of all variables related to the scientific and technical parameters of the studies referred in the research applications evaluated were associated with a higher ratio of obtaining a successful ethical review. These findings indicate that a favorable opinion was not dependent on the characteristics of the study. However, the findings confirm an association between deficiencies in sensitive aspects of the research application, such as documentation submitted, informed consent, research objectives and hypotheses, methods and procedures, legal, and economic reports, and a non-favorable opinion in the first ethical review process. On one hand, these findings indicating deficiencies in the applications are in consonance with previous studies in which similar outcomes have been reported, such as: incomplete documentation (24), legal and ethical aspects inappropriately addressed (2, 25), informed consents not properly written (2, 19, 26), and an insufficient description of sensitive aspects related to the objectives, methods or procedures (2, 19). In addition, some of the aforementioned aspects have been associated either with a non-favorable opinion or with delays in the ethical review process (25). On the other hand, findings reported in relation with the PI's profile indicate some important aspects that require a separate consideration. Having research as a main professional activity and having previous experience submitting research applications appear associated with a greater chance of obtaining a positive opinion in the review process. These evidences confirm the importance that targeted training and experience have in the preparation of research applications with a high scientific quality. Unfortunately, information such as the PI's age or years of professional experience were not collected in this study. This type of information could allow a deeper analysis of this matter. However, the higher ratio of successful observed in applications written by PIs with a leadership working position (PIs who are research group leaders or heads of department), offers evidence supporting the positive impact that having experience and being trained has for researchers who assume the role of a PI. In addition, being a male also appear as a variable associated with a higher success rate. This finding, more than an indication of a difference related to gender, should be interpreted as a direct consequence of the fact that most leadership positions are occupied by male researchers, this being confirmed in the logistic multivariable analysis in which this variable was dismissed. Neither being a professional-in-training (which implies having the support of a supervisor or a mentor), having a doctoral or PhD degree, being a clinician, nor working in collaboration with other colleagues were associated with a higher success ratio. These findings bring more evidence supporting the important role that targeted training and research experience have in the preparation of a robust research application. However, these findings are also in consonance with the need that a researcher who leads a research application should have sufficient research experience and qualification or alternatively collaborate with more experienced colleagues in the field of his/her research, as has been recently stated by Beshir (9). In fact, the lack of training and experience of the research group has been described as one of the main causes of failures in research applications submitted for a review process in other European countries (2, 24).

The last aim of this study was to determine which of the aforementioned variables with statistical association appeared as predictors of a favorable opinion at the first ethical review. A multiple logistic regression confirmed that only three variables appeared as predictors in a model explaining 71% of the probability of obtaining a favorable opinion. Two of these variables are associated with sensitive aspects of the research application, such as having an informed consent and methods and procedures properly written and adequately explained. These findings are in accordance with the majority of the evidence reported, where both elements are described as the most frequently cause of rejections and delays in ethical review processes (2, 19, 25, 26). Having a leadership position appears in the aforementioned model as the third predictor of a favorable opinion in the first ethical review process confirming the main hypothesis of this study. This finding not only brings new evidence supporting the important role that a targeted training and experience in research play in this matter, which is in accordance with the opinion of other authors and the evidence reported (2, 9). This finding also provides strong evidence supporting the importance that leadership and mentoring have in interdisciplinary teams performing research activities. In particular, this finding focuses on the specific role of the senior researcher who holds a leadership position in their group or department where these research activities are carried out. This is consistent with the contribution made by other authors (27–29) establishing a direct relationship between leadership and mentoring performed by the most experienced researchers and the improvement in indicators of scientific productivity in their teams. Those indicators include, for example, research projects with high quality, publications, or the consolidation of the scientific careers of less experienced researchers. In the context of professionalism as a paradigm of “job well done” in biomedical research, it is desirable that this third predictor disappeared in the future. Because it suggests, in worst case scenarios, the existence of research groups with very hierarchical compositions, where high experienced group leaders are not transmitting their knowledge and experience to less experienced researchers. In this frame, the reinforcement of lifelong learning and inter-professional collaborative abilities could be two successful strategies for addressing this gap.

### Limitations

This study included a heterogeneous group of studies with different methodologies, study designs, and researchers from different academic and professional backgrounds and profiles. In addition, the study covered only studies submitted to one Research Ethics Committee for an ethical review. Due to the nature of the Autonomous Community of La Rioja, the majority of the studies submitted corresponded to only one healthcare institution, the University Hospital San Pedro of La Rioja. Considering the complexity of the phenomenon analyzed, it is recommended that other two aspects should be included in further studies, such as the age of the PIs or their years of research experience. Unfortunately, both aspects were not collected in this study. However, findings reported in this study bring novel evidence that can help our understanding of elements influencing in a favorable opinion in the review process. Future lines of research could focus in a deep analysis of some of the factors described in this study.

In conclusion, in the wide context of a “job well-done” the aforementioned findings bring new evidence supporting the importance that professionalism plays in biomedical research. In the specific case of biomedical research studies involving human subjects, it implies preparing research applications fulfilling adequate scientific, methodological, legal and ethical standards of quality. In this sense, Research Ethics Committees play an important role as guarantors that such standards are complied before they are executed. Therefore, research applications with deficiencies in some of these aspects are in risk of receiving a non-favorable opinion once they are submitted for a first ethical review or of having delays in obtaining a favorable opinion. Researchers in charge of the preparation of research applications should pay attention to bringing a complete information of the study design, methods and procedures according to the applicable normative in order to ensure a favorable review process. In this frame, more-experienced researchers holding leadership positions in their research groups play a fundamental role during the preparation of new research proposals. Based on the evidence reported in this study, it is recommendable that research group leaders enhance the improvement of lifelong learning and inter-professional collaborative abilities in their teams in order to reduce training gaps.

## Data Availability Statement

The raw data supporting the conclusions of this article will be made available by the authors, without undue reservation.

## Ethics Statement

The studies involving human participants were reviewed and approved by Research Ethics Committee of the Foral Community of Navarra (Ref. PI 2021/57). Written informed consent for participation was not required for this study in accordance with the national legislation and the institutional requirements.

## Author Contributions

LV was in charge of the study's overall design. EM, LG-A, MTA-G and BB were in charge of data collection. LV and MS-M performed the statistical process of the data. LV, EM and RCDB prepared the draft manuscript. All authors contributed to the present work, participated in the interpretation and processing of results, and reviewed and approved the final manuscript.

## Funding

This work was economically supported by the Call for Aids Financed by Donations for Projects Related to SARS-CoV-2 Infection of the Autonomous Government of La Rioja, Spain (Ref. COV21-DONAC1/id.proy.1110), and by the Rioja Health Foundation (Ref. FRS-CEIMLAR-2022).

## Conflict of Interest

The authors declare that the research was conducted in the absence of any commercial or financial relationships that could be construed as a potential conflict of interest.

## Publisher's Note

All claims expressed in this article are solely those of the authors and do not necessarily represent those of their affiliated organizations, or those of the publisher, the editors and the reviewers. Any product that may be evaluated in this article, or claim that may be made by its manufacturer, is not guaranteed or endorsed by the publisher.
